# Measurements of Rationality: Individual Differences in Information Processing, the Transitivity of Preferences and Decision Strategies

**DOI:** 10.3389/fpsyg.2017.01844

**Published:** 2017-10-18

**Authors:** Patrycja Sleboda, Joanna Sokolowska

**Affiliations:** Faculty of Psychology, SWPS University of Social Sciences and Humanities, Warsaw, Poland

**Keywords:** dual-process theories, decision strategies, transitivity of preferences, bounded rationality, indexes of rationality

## Abstract

The first goal of this study was to validate the Rational-Experiential Inventory (*REI*) and the Cognitive Reflection Test (*CRT*) through checking their relation to the transitivity axiom. The second goal was to test the relation between decision strategies and cognitive style as well as the relation between decision strategies and the transitivity of preferences. The following characteristics of strategies were investigated: requirements for trade-offs, maximization vs. satisficing and option-wise vs. attribute-wise information processing. Respondents were given choices between two multi-attribute options. The options were designed so that the choice indicated which strategy was applied. Both the *REI-R* and the *CRT* were found to be good predictors of the transitivity of preferences. Respondents who applied compensatory strategies and the maximization criterion scored highly on the *REI-R* and in the *CRT*, whereas those who applied the satisficing rule scored highly on the *REI-R* but not in the *CRT*. Attribute-wise information processing was related to low scores in both measurements. Option-wise information processing led to a high transitivity of preferences.

## Introduction

In research on decision-making much attention during last few decades has been devoted to personal information processing styles. This interest is related to the growing popularity of Dual-Process Theories (*DPT*) in which two cognitive systems are emphasized. The first is intuitive, quick, unconscious and affect-based, whereas the second is logical, conscious, slow and reason-based, also labeled referred as “rational” (e.g., Epstein, 1994; Kahneman, 2003). Various measurement techniques have been developed to diagnose the personal style of information processing. Two classes of these measurements can be distinguished: self-reported inventories and task solving tests. In the study presented here, both types of measurements were used, i.e., self-reported Rational-Experiential Inventory (the *REI*) proposed by Pacini and Epstein (1999) and the Cognitive Reflection Test (the *CRT*) developed by Frederick (2005).

The important contribution from the present research is that these measurements of information processing have been confronted with the fundamental assumption of rational choices accepted in utility theory (*UT*), i.e., the transitivity of preferences (Von Neumann and Morgenstern, 1953). Despite a high interest in personal styles of information processing in decision research, to our knowledge neither measurement of *DPT* has been directly confronted with the concept of transitivity of preferences in *UT*^1^. So the first goal of this study was to validate two *DPT* measures of rationality: the *REI* and the *CRT* in light of the transitivity of preferences, the measure of rationality in *UT*.

The second goal of the study is to investigate the influence of individual differences in information processing on the strategies applied to selections among multi-attribute options. The following properties of strategies were investigated: requirements for trade-offs (compensatory vs. non-compensatory), maximization vs. satisficing and option-wise vs. attribute-wise information processing. These properties were selected because they allow differentiating among decision strategies related to the classical concept of rationality, to the concept of bounded rationality and to simple heuristics. It was expected that high scores on the Rationality subscale of the *REI* (the *REI-R*) and in the *CRT* would be related either to option-wise compensatory strategies and to maximization or to option-wise non-compensatory strategy and to satisficing. In contrast, low scores on the *REI-R* and in the *CRT* would be related to dimension-wise non-compensatory strategies^2^.

The third goal was to test whether the strategies based on option-wise information processing favor the transitivity of preferences, independently of the requirement of trade-offs. Therefore, another important contribution from the presented research is the test of preferences' transitivity in the light of information processing behind various decision strategies.

The distinction between two means of cognition, emphasized within *DPT*, is not a new idea. Immanuel Kant (1781) introduced the concept of *a posteriori* and *a priori* judgments. *A posteriori* judgments are derived from experience and are based on *phenomena* that are related to sensation. These judgments don't lead to accurate representations of objects “as they are in themselves” and relations between them. In contrast, *a priori* judgments are derived from analytical thinking, or are synthetic a priori truths based on abstract reasoning *noumena*, i.e., objects as they are in themselves, independently of the senses. The concept of extensional and intuitive reasoning, introduced by Tversky and Kahneman (1983), is very similar to Kant's theory. This is also the fundamental concept in *DPT* proposed by Kahneman (2003; Kahneman and Frederick, 2005) who emphasized two cognitive systems: (1) intuitive, quick, unconscious, affect-based and (2) logical, conscious, slow, reason-based.

Frederick (2005) developed the *CRT* to probe individual differences in the dominant information processing system. The *CRT* consists of three mathematical questions (e.g., *In a lake*, the*re is a patch of lily pads. Every day*, the *patch doubles in size. If it takes 48 days for* the *patch to cover* the *entire lake, how long would it take for* the *patch to cover half of the lake?*). To answer these questions correctly one has to use System 2. On the other hand, wrong answers produced by System 1 come to mind automatically. High performance in the *CRT* means that System 2 “wins” with System 1 and indicates ability for analytical thinking. It was shown in number of studies that score in the *CRT* correlates positively with scores in IQ tests, SAT total score (Frederick, 2005; Obrecht et al., 2009; Toplak et al., 2011; Liberali et al., 2012), the ability to delay gratifications and the percentage of risky choices consistent with the normative model (Frederick, 2005). Similarly, Cokely and Kelley (2009) found that participants who scored highly in the *CRT* made choices in line with expected value. At the same time, a low score in the *CRT* positively correlated with the answers that reflected the conjunction and gambler's fallacies, neglect of base rate and sample size, framing and more (Toplak et al., 2011). The *CRT* also measures numeracy skills, i.e., the skills that enable understanding and number use (e.g., Frederick, 2005; Reyna et al., 2009). In light of *DPT*, the most important property of the *CRT* is that the score in the *CRT* correlates with the Stroop effect (Stroop, 1935), as shown by Toplak et al. (2011). This indicates that analytical information processing with the aid of System 2 requires conscious overcoming of automatic responses evoked by System 1. Therefore one may conclude that the *CRT* is a good measure of poor vs. high information processing.

Alternatively, Pacini and Epstein (1999) proposed a self-reported inventory, the *REI* (the Rational-Experiential Inventory), to identify individual differences in information processing. This inventory consists of two subscales: *Experiential* (the *REI-E, “I like to rely on my intuitive impressions”*) and *Rational* (the *REI-R, “I enjoy problems that require hard thinking”*). The *REI-R* has been found to be a strong predictor of performance consistent with the normative rules in tasks such as missing-a-flight vignette, the thematic and abstract versions of Wason task and the jelly bean task (Witteman et al., 2009). Ayal et al. (2011; Ayal et al., 2012) reported that participants, who scored high on the *REI-R* less frequently chose low-diversified portfolios and less frequently had reversed preferences for normatively identical options in two different situations. On the basis of the above findings, one may conclude that a high score on the *REI-R* is a good predictor of rational behavior.

In light of the cited findings, it is not surprising that the scores on the *REI-R* and in the *CRT* are positively correlated (Liberali et al., 2012; Thoma et al., 2015). On the other hand, these two measurements reflect two different methodological approaches: the score in the *CRT* results from actual performance, while the *REI* is a self-declarative measurement. Thus, it was interesting to check how both measurements account for transitivity of preferences and for strategies applied for selection among multi-attribute options.

Deviations in actual behavior from the fundamental assumptions of rational choices accepted in *UT* (Von Neumann and Morgenstern, 1953) have been studied for many years (e.g., Edwards, 1954; Simon, 1955, 1972, 1978, 1986; Kahneman and Tversky, 1972, 1973; Tversky and Kahneman, 1973, 1974, 1983; Shafir et al., 1993) and much attention has been paid to deviations from transitivity of preferences (e.g., May, 1954; Tversky, 1969). The transitivity axiom requires a systematic order of preferences across options. This means that for any three options A, B, and C, if A is preferred to B and B is preferred to C than A has to be preferred to C. The assumption of preference transitivity is the basic requirement of rational choice (Fishburn, 1991; Müller-Trede et al., 2015). Since in *DPT* rationality is related to information processing with the aid of System 2, there should be a relation between measures of the dominant style in information processing and the transitivity of preferences. So in the present study we checked whether *DPT* measurements account for transitivity of preferences.

**Hypothesis 1:** High scores on the *REI-R* and in the *CRT* are related to high transitivity of preferences.

The second goal of the study was to investigate the influence of individual differences in information processing on strategies applied in selection among multi-attribute options. In the light of information processing, the following properties of decision strategies are important: requirements for trade-offs (compensatory vs. non-compensatory), maximization vs. satisficing and option-wise vs. attribute-wise information processing.

As for the trade-offs' requirement, compensatory and non-compensatory decision strategies can be distinguished. The basic compensatory strategy is the Multi-Attribute Utility (*MAU*) model. According to this strategy, the decision maker estimates the score (utility) on all the attributes and assigns weights to them. The utility of each attribute is multiplied by its weight. The option with the highest weighted sum should be chosen. There are also other linear models that are less sophisticated, e.g., Franklin's Rule, Tallying (Gulliksen, 1950), but all of them require: (1) linear integration of information, (2) inter-dimensional trade-offs, and (3) a separate, global evaluation of each option. Does such a model always lead to rational choices? Imagine that one has to evaluate the health of three individuals considering three essential, equally important organs, as described in Table 1. According to the linear model, Person 2 has the highest weighted sum and, therefore, should be considered the healthiest. Yet clearly we are not able to survive without any one of the above organs. From this perspective, the healthiest is Person 3, even though s/he has the lowest global evaluation. Is choosing Person 3 an irrational decision? A solution to the above problem may be the strategy that doesn't require trade-offs, as proposed by Simon (1955, 1972, 1978). It still requires option-wise evaluations, but the optimization criterion is relaxed. According to Simon's concept of Bounded Rationality (Simon, 1957), people make decisions that may not be optimal but are still satisficing. Simon's idea is that it is sufficient to pick an option that satisfies aspirations The decision maker searches through options only until one that meets aspirations is found. So instead of the maximization of additively integrated utilities from all attributes, the comparison of scores for these attributes with the satisficing threshold is the criterion for choice (conjunction rule—*CON*).

**Table 1:** Three options described on three attributes.

	**Weights**	Option 1^*^	Option 2^**^	Option 3^***^
Heart	0.33	8	1	5
Lungs	0.33	10	10	5
Liver	0.33	1	10	5
Weighted sum		6.27	6.93	4.95

* *Choice of Option 1, assuming heart to be the most important attribute, is in line with Lexicographic (LEX) strategy*.

** *Choice of Option 2 is in line with Multi-Attribute Utility (MAU) strategy*.

*** *Choice of Option 3, assuming 5 as a minimum cutoff, is in line with Conjunctive (CON) strategy*.

The most important difference between *MAU* and *CON* is that they imply different criteria for decision: either averaging or meeting minimal cutoffs on all the relevant attributes. On the other hand, applying either strategy requires the systematic processing of all relevant information, which entails relatively high cognitive effort. This can be avoided if one uses another class of non-compensatory strategies, i.e., lexicographic strategies, which are much simpler than *CON*.

In these strategies, options are not treated separately but compared on the basis of attributes in the following way: (1) the decision maker orders attributes by importance and (2) chooses the option that is the best on the most important attribute, e.g., Person 1 from Table 1, if healthy heart is the most important attribute. If options are equally good on the most important attribute, the second most important attribute is considered, etc. There are many non-compensatory models based on attribute-wise comparisons such as Lexicographic) Rule (*LEX*, e.g., Luce, 1956; Tversky, 1969; Fishburn, 1970; Luce et al., 2000), Elimination by Aspects (Tversky, 1972), Minimax, Take-The-Best heuristic^3^ (Gigerenzer and Goldstein, 1996) or Priority Heuristic (Brandstatter et al., 2006). Even though lexicographic models differ among themselves, all of them are based on the same idea that choices are made on the basis of comparative judgments and incomplete, selective information processing. This is in contrast to *MAU* and *CON* strategies, where choices are based on global evaluations that include all the relevant information, made independently for each option.

The relation between applied strategies and information processing as well as transitivity of preferences requires explanation. It follows from previous research that people who base their decisions predominantly on System 1 are focused on perceptual features of a task (Sloman, 1996; Kahneman, 2003) and are prone to the biases that stem from context-dependent judgments (Payne et al., 1988, 1993). Such people therefore either use simple lexicographic rules or switch between different strategies. On the other hand, focusing on abstract aspects of a problem protects one against such judgmental biases. As mentioned earlier, Ayal et al. (2011) found that people who scored highly on the *REI-R* were less prone to reverse preferences for normatively identical options set in different contexts. So one may conclude that people with dominant System 2 consider all relevant aspects and consistently apply the same criterion for similar choices. For example, a person may consistently believe that either averaging (*MAU*) or meeting aspirations (*CON*) leads to the most accurate decision.

**Hypothesis 2:** People who score highly on the *REI-R* and in the *CRT* more frequently apply strategies based on option-wise information processing, i.e., either *MAU* type or *CON* strategies.

As for transitivity, the common view is that lexicographic rules lead to intransitivity of preferences, as a result of shifting attention among various dimensions. Since some information is ignored, judgment is partial. Tversky (1969) proposed a different explanation of intransitivity. He assumed that people differ not only in the relative importance assigned to various attributes but also in Just Noticeable Difference (*JND*) in scores across various attributes. He gave the example of three job candidates: *X, Y, Z*, who differed with respect to two attributes: intelligence and experience. Under the assumption that IQ is more important than experience, the candidate who scores higher in IQ than the others should be chosen. If such a difference, however, is not salient, one will look at differences in experience. This may lead to intransitive preferences, when the difference in IQ between *X* and *Y* as well as between *Y* and *Z* is not salient and then they are ordered due to the other dimension that points to *X*. On the other hand, the difference between *X* and *Z* in IQ scores may be noticeable—thus *Z* is chosen over *X*—resulting in intransitive preferences. This structure is called a lexicographic semi-order (*LS*) that results in intransitivity, “*where a semi-order (LS)* (Luce, 1956) *or a just noticeable difference structure is imposed on a lexicographic ordering*” (Tversky, 1969, p. 32).

The important feature of Tversky's explanation is that this can be extended to intransitivity that results from additive models. He gave an example of the Additive Difference (*ADD*) model. In the *ADD*, differences in scores for each dimension are calculated for both options and then these differences are summed. Differences in *JND* for various attributes can lead to the intransitivity of preferences. Note, *LS* can be treated as a specific case of *ADD*—where at least one difference in scores is described as a step function. So both models, *ADD* and *LS*, can result in intransitive preferences, when differences in scores are described by non-linear functions.

From Tversky's reasoning (1969), it follows that the compensatory principle does not prevent the intransitivity of preferences, when sensitivity to various dimensions is described by different non-linear functions and judgment is comparative. The important question is whether the linearity requirement is necessary, when information processing is option-wise and then each option is evaluated independently as in *MAU* and in *CON* strategies. If the utility of scores is defined by a monotonic function, one may expect transitive orderings of independently formed global evaluations. For *MAU* it is assumed that the higher the score, the higher its utility. For *CON* one can assume that all utilities of scores are described by step functions. Therefore, **Hypothesis 3** was formulated as follows:

**Hypothesis 3:** Choices based on *MAU* or *CON* strategies result in transitive preferences.

## Materials and methods

### Overview of experimental design

Hypotheses 1–3 were verified in the study whereby respondents chose between two options constructed such that the selected option indicated the applied strategy (*MAU, CON, LEX*). Options were described three common attributes. To investigate the relation between individual differences in information processing and applied decision strategies, one should minimize the impact of situational factors. This can be achieved when people solve abstract problems, where there are no interferences with the previous experience, familiarity, or emotions. An abstract presentation was also used in order to diminish the influence of content on the salience/perceived importance of attributes (Gallhofer et al., 1987; Sokolowska, 1990). Another important characteristic of abstract choices is that there are no differences in JND across attributes. One may also assume that scores' utility function for abstract attributes is monotonic. So in the present research, respondents were faced with identical choices set either in abstract or in specific context.

Respondents in one group were choosing one of two apartments described on attributes that had specific modalities (specific content). In contrast, participants in the second group were choosing between two abstract options that were described on three common dimensions that had no specific modalities (abstract content).

Beside the influence of the content, decision makers may also be influenced by the goal that s/he wants to achieve. Maximizing choice accuracy is not necessarily the goal of all decision makers. Other goals might be also justified, such as minimization of effort, regret or conflict (Einhorn and Hogarth, 1981; Tetlock, 1985). For example, if minimizing effort rather than maximizing accuracy is the goal, one may use the easiest *LEX* strategy. In order to unify the goals of respondents, financial rewards were introduced for those who made the most accurate choices.

### Participants

Two hundred and nine respondents voluntarily participated in this study, conducted online. In Group 1 (choice of apartment) there were 105 respondents (53.3% female, *M* age = 27.9; *SD* = 5.71). 61.9% of the participants had master degree, 8.6% bachelor degree, 21% were students. In Group 2 (abstract choice) there were 104 participants (481% female, *M* age = 29.4; *SD* = 6.71). 66% of the participants had master degree, 13.5% bachelor degree and 13.5% were students.

To motivate them, participants were told that the three participants in each group who achieve the highest number of correct choices, would get 300 PLN (80 USD) gift card to a bookstore. Respondents were told that one option was always better, however, accuracy here was not defined. Financial rewards were given to those who had the highest number of choices made in line with *MAU* model, although participants were not aware of this.

### Task

**Figure 1:**
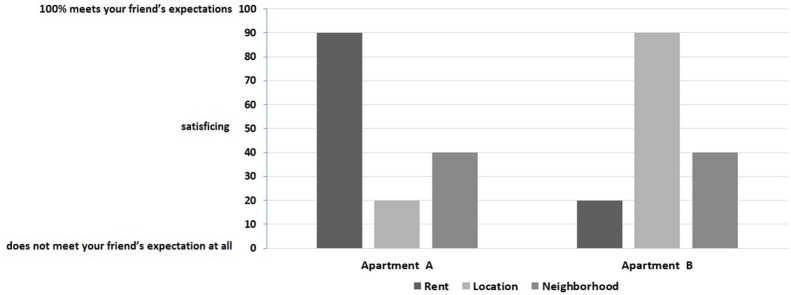
Group I: An example of one out of 26 choices.

Participants in Group 1 (specific content) were to imagine that they had been asked by a friend to help in choosing one of two apartments. They were told that their friend had already evaluated each apartment in terms of three attributes—rent, location and neighborhood—which s/he considered the most important and of equal importance (see Figure 1).

Participants in Group 2 (abstract content) made choices between two abstract options that had no content and where the attributes had no modalities. In particular, participants were given the following instruction: “*You will be faced with series of choices that have no specific content. It does not matter whether you choose between two cars, two kinds of washing powder, two schools, two jobs or two candidates for the president of a country, etc. We decided to present you with abstract choices rather than with the concrete ones because we are interested in general principles of judgment and choice processes that should not depend on the object of choice. Imagine that you were asked by a friend to help choose between two options described in terms of the three most and equally important attributes……”*

Respondents in both groups were told that their task would be to help a decision maker choose better one of two options. Each respondent was to make 26 choices between two options, always described in terms of the three attributes. The choice options were identical in both groups and the only difference was that in Group 2, neither the options nor the attributes had concrete modalities. Stimuli were presented as histograms. The scores for each attribute were presented as bar heights; the higher the bar, the better the score.

To assure that utility of scores on attributes would be described by a monotonic function (see p. 11), the scores on all attributes were given in numbers from 0 to 100 with assigned labels such as 0 the worst score, 50 the satisfactory score and 100 the maximum score. It was expected that in the abstract task respondents should treat scores as described either by linear or by step functions.

### Measurements

#### The REI-short 24^4^ and the CRT

The short, 24-item *REI* (Pacini and Epstein, 1999), previously validated by Ayal et al. (2012; Ayal et al., 2011) and Ayal et al. (2015), was translated into Polish by Sleboda and Sokolowska. The reliability of the *REI* questionnaire was checked for both subscales. For the *REI-R*, Cronbach's alpha coefficient was α = 0.79. Cronbach's alpha coefficients were similar in both groups (α = 0.78 and α = 0.81 for specific and abstract content, respectively). For the *REI-E* α = 0.84 for all respondents and α = 0.85 in Group 1 and α = 0.84 in Group 2. The correlation between the two subscales was negative (for Group 1: *r* = −0.237; *p* = 0.02, *N* = 105; for Group 2: *r* = −0.014; *p* = 0.89, *N* = 104; for both studies: *r* = −0.12; *p* = 0.08, *N* = 209). The difference in scores on both subscales between studies was not significant [*F*_(1, 207)_ = 0.062 and 0.002, *p* = 0.80 and 0.97, η_p_^2^ = 0.000 for the *REI-R* and the *REI-E* respectively]. Since the focus of the present studies is on rationality, only the *REI-R* was considered in further analysis.

The respondents also answered the *CRT* questions (Frederick, 2005). 32.1% of respondents answered all the *CRT* questions incorrectly, while 26.3% answered all questions correctly. The difference in distributions of answers between participants who chose an apartment and those who made abstract choices, was insignificant (χ2 = 2.72, *df* = 3, *N* = 209, *p* = 0.44).

Since the *REI* and the *CRT* are two types of techniques to distinguish the dominant information processing system, one may expect a positive correlation between the *REI-R* and the *CRT* scores. Indeed, these two scores were significantly positively correlated for all respondents (*r* = 0.33, *N* = 209, *p* < 0.001) as well as for specific (*r* = 0.30, *p* = 0.002, *N* = 105) and for abstract (*r* = 0.36, *p* < 0.001, *N* = 104) content.

#### Transitivity

Seven sets of three choices, e.g., choices 4, 13, 19 (see Table 2), allowed us to test the transitivity axiom.

**Table 2:** Options used in 26-choices design to differentiate among strategies.

**Choices that differentiated between:**	**Choice no**	**Option 1**	**Option 2**
*LEX* vs. *CON*	4	90:20:40	50:50:50
	5	90:40:20	50:50:50
	6	40:90:20	50:50:50
	7	20:90:40	50:50:50
	8	20:40:90	50:50:50
	9	40:20:90	50:50:50
*MAU* vs. *CON*	13	70:30:60	50:50:50
	14	70:60:30	50:50:50
	15	60:70:30	50:50:50
	16	30:70:60	50:50:50
	17	30:60:70	50:50:50
	18	60:30:70	50:50:50
*MAU* vs. *LEX*	19	70:30:60	90:20:40
	20	70:60:30	90:40:20
	21	60:70:30	40:90:20
	22	30:70:60	20:90:40
	23	30:60:70	20:40:90
	24	60:30:70	40:20:90
Additional choices	1	90:20:40	20:90:40
	2	90:40:20	20:40:90
	3	40:90:20	40:20:90
	10	80:30:60	30:70:60
	11	70:60:30	30:60:80
	12	60:80:30	60:30:70
	25	90:20:40	60:60:60
	26	70:30:60	60:60:60

If there are three options: A, B, and C, and all three pairwise choices (A/B, A/C, and B/C) are given to participants, then there are six combinations that are consistent with the transitivity axiom. These combinations are:

1. if A>B and B>C then A>C,
2. if C>B and B>A then C>A,
3. if B>A and A>C then B>C,
4. if C>A and A>B then C>B,
5. if A>C and C>B then A>B,
6. if B>C and C>A then B>A.

For example, for a set of choices 4, 13 and 19, three options scored as follows on three attributes: A = 90,20,40; B = 50,50,50 and C = 70,30,60. The corresponding sets of transitive choices were as follows:

1. if (90,20,40) > (50,50,50) and (50,50,50) > (70,30,60), then (90,20,40) > (70,30,60),
2. if (90,20,40) < (50,50,50) and (50,50,50) < (70,30,60), then (90,20,40) < (70,30,60),
3. if (90,20,40) < (50,50,50) and (90,20,40) > (70,30,60), then (50,50,50) > (70,30,60),
4. if (90,20,40) > (50,50,50) and (90,20,40) < (70,30,60), then (50,50,50) < (70,30,60),
5. if (90,20,40) > (70,30,60) and (70,30,60) > (50,50,50), then (90,20,40) > (50,50,50),
6. if (50,50,50) > (70,30,60) and (90,20,40) < (70,30,60), then (90,20,40) < (50,50,50).

Independently of the preferred strategy, a participant had to choose one of these six combinations to be transitive. Any other choice was inconsistent with the transitivity axiom. For each transitive choice, a participant was given one point, otherwise zero. Since there were 7 sets to test transitivity, each participant could score from 0 to 7 points. A vast majority of respondents scored between 5 and 7 (the maximum) in the transitivity index. Only 1% (2 participants) scored 3, and 2% (4 participants) scored 4. They were excluded from further analyses since their groups were too small to compare with others. The observed high score in transitivity is in agreement with the expectation that people obey the transitivity axiom when the utility of scores is defined by a monotonic function (see p. 11).

51.7% of participants always obeyed the transitivity axioms. More participants scored 7 in the transitivity index in abstract choices (56.7%) than in specific choices (46.7%). The average index of transitivity was also higher for abstract choices [*F*_(1, 202)_ = 4.18, *p* = 0.04, η_p_^2^ = 0.020]. This, again, is in agreement with expectations that in abstract choices *JND* in utility across attributes does not differ, because this utility is most likely described by a linear function.

#### Strategies applied for the selections

Twenty six choices were constructed to determine which strategy was applied: *MAU, CON* (*satisficing*), or *LEX*^5^. The choices considered by respondents are presented in Table 2.

As can be seen from Table 2, 18 choices were designed specifically to differentiate among strategies and to assign either *MAU* or *CON* strategy. For example, to differentiate between *CON* and *MAU*, Choices 13–18 where used, where Option 1 always has a greater sum of weighted scores than Option 2. Another six, Choices 4–9, were used to differentiate between *CON* and *LEX*. Here, the sum of weighted scores is the same. In all 12 choices, only in Option 2 all dimensions meet the satisfactory level. To decide whether a respondent used *CON*, we summed up all choices in line with *CON* and we accepted the criterion of 10 out of 12 choices to assign this strategy to the respondent. The 10 choices out of 12 were accepted on the basis of a comparison of the probability of this sequence when a given strategy is applied with the probability of the sequence appearing randomly. The detailed description of the way in which strategies were assigned is given in Appendix 1 (Supplementary Material), accepted for assigning a strategy.

To assign the *MAU* strategy the same analysis was done for Choices 13–18 (that distinguish *MAU* from *CON*) and Choices 19–24 (that distinguish *MAU* from *LEX)*.

For assigning *LEX* strategy, the procedure was extended to two steps. In the first step, it was decided which attribute was the most important on the basis of three combinations of Choices 1, 2 and 3. For example, the choice of Option 1 in Choice 1 and in Choice 2 indicated that the first attribute was most important. Next, for each attribute an additional 10 choices were analyzed (12 in total). For example, for Attribute 1, the choices were as follows: 4, 5, and 25 (Option 1), 19, 20, and 11 (Option 2), 13 and 14 (Option 1), 16 and 17 (Option 2). The same pattern was used for the two other attributes.

Respondents were assigned to group *LEX1*, if they had series of 10 out of 12 choices on Attribute 1, *LEX2* if they had series of 10 out of 12 choices on Attribute 2 and *LEX3* if they had series of 10 out of 12 choices on Attribute 3. Finally, the respondents from these 3 groups were put to one labeled *LEX*. For 4 participants, it was not possible to differentiate between the *LEX* and *MAU* strategies. These respondents were excluded from further analysis.

The results of strategy assignment are given in Table 3.

**Table 3:** Classification of subjects on the basis of strategies applied for selection.

**Strategy**	**Number of participants to whom a given strategy is attributed**	
	**Group 1**	**Group 2**
Inconsistent	37	40
*LEX*	20	8
*CON*	18	21
*MAU*	26	35
Total	101	104

As can be seen from Table 3, a majority of participants was consistent in using the same criterion for choice. The most salient difference between the two groups was that 19.8% of those who chose an apartment used *LEX*, whereas only 8 participants (excluded from further analyses) applied an *LEX-like* rule for abstract choices. 25.7% of participants in specific and 36.5% in abstract content consistently chose according to the *MAU* strategy. 17.8 and 21.9% of participants consistently used *CON* in specific and abstract choices, respectively. The differences between groups were significant (χ2 = 21.56, *df* = 3, *p* < 0.001).

## Results

### The relation between the *REI*, the *CRT* and the transitivity of preferences

In order to check the relations between *DPT* measurements and transitivity, the process regression Model 1 (Preacher and Hayes, 2008; Hayes, 2009) with transitivity as the dependent variable was employed with 1,000 bootstrap samples. This technique was used because it allows one to check the conditional effect of content which is a dichotomous variable. Because the *REI-R* and *CRT* were positively, significantly correlated, this analysis was performed for each measurement technique separately.

The score on the *REI-R* (as an independent factor) was a good predictor of preference transitivity in abstract choices (0.036, *SE* = 0.012, *p* = 0.004, the lower 0.012 and upper 0.061 bounds) but not for specific ones (−0.002, *SE* = 0.014, *p* = 0.01, the lower −0.029 and upper 0.026 bounds).

A similar result was obtained for the *CRT* as the independent factor and transitivity as the dependent variable. The score in the *CRT* was positively correlated with the transitivity of preferences for abstract choices (0.181, *SE* = 0.070, *p* = 0.011, the lower 0.043 and upper 0.320 bounds) but not for specific ones (−0.057, *SE* = 0.069, *p* = 0.41, the lower −0.192 and upper 0.079 bounds)^6^.

The insignificant correlations between *DPT* measurements and transitivity in specific content might have been caused by the difference in strategies applied in each context. This is discussed in the next section.

### Individual differences in information processing and applied decision strategies

#### The applied strategy and the score on the REI-R

**Figure 2:**
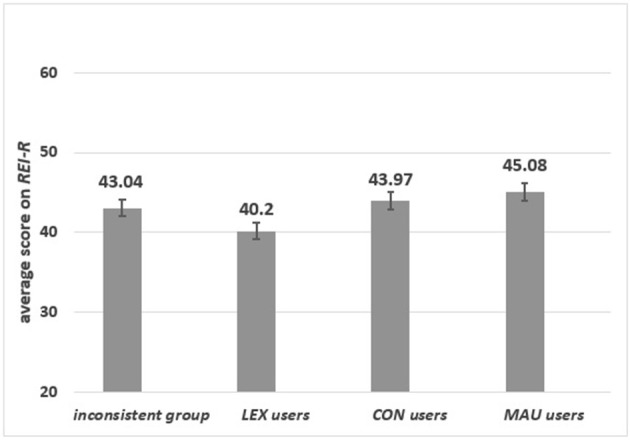
The average scores on the *REI-R* for respondents who applied various strategies.

As can be seen from Figure 2, there were significant differences among users of specific strategies in the score on the *REI-R* [*F*_(3, 193)_ = 3.40, *p* = 0.02, η_p_^2^ = 0.050]. *LEX*-users scored the lowest on the *REI-R*. In contrast, those using either *MAU* or *CON* strategies and the inconsistent group scored equally high on the *REI-R*. There was no significant difference between *MAU* and *CON*-users (*p* = 0.39), while a marginally significant difference was observed between *MAU*-users and the inconsistent group (*p* = 0.06).

*Group 1: Specific content*. Significant differences in the scores on the *REI-R* were found [*F*_(3, 97)_ = 2.92, *p* = 0.04, η_p_^2^ = 0.083] for *MAU, CON, LEX* users and the inconsistent group. As presented in **Figure 4**, *LEX*-users scored the lowest on the *REI-R* and this group differed significantly from *MAU*-users (*p* = 0.02), *CON*-users (*p* = 0.02) and from the inconsistent group (*p* = 0.01). There were no significant differences among the other groups.

**Figure 3:**
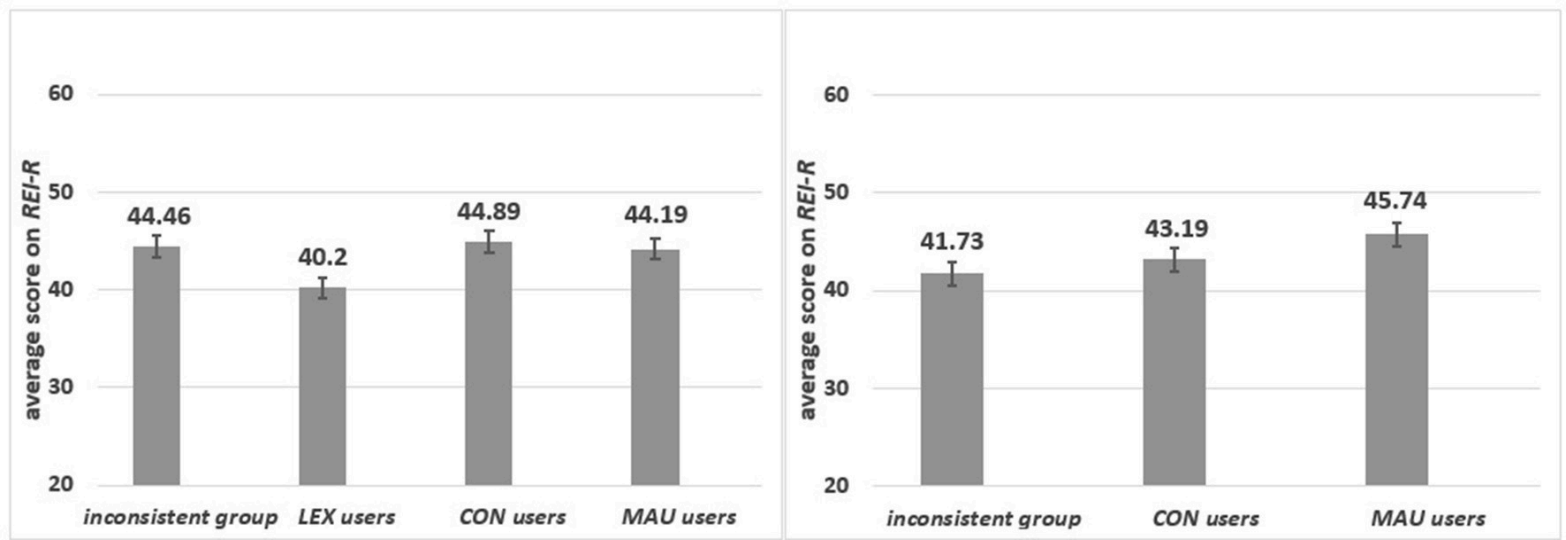
The average scores on the *REI-R* for respondents, who applied different strategies in specific **(left panel)** and in abstract **(right panel)** content.

*Group 2: Abstract content*. The significant differences in the scores on the *REI-R* were observed [*F*_(2, 93)_ = 3.56, *p* = 0.032, η_p_^2^ = 0.071] among users of specific strategies^7^. There were significant differences between *MAU*-users and the inconsistent group (*p* = 0.009), whereas *CON*-users did not differ significantly from either *MAU*-users (*p* = 0.160) or from the inconsistent group (*p* = 0.407) (see Figure 3).

In summary, participants with high scores on the *REI-R* consistently used strategies in which all relevant information was used. However, with specific content, the inconsistent group scored as high as *CON* and *MAU*-users.

#### Strategy and the CRT

**Figure 4:**
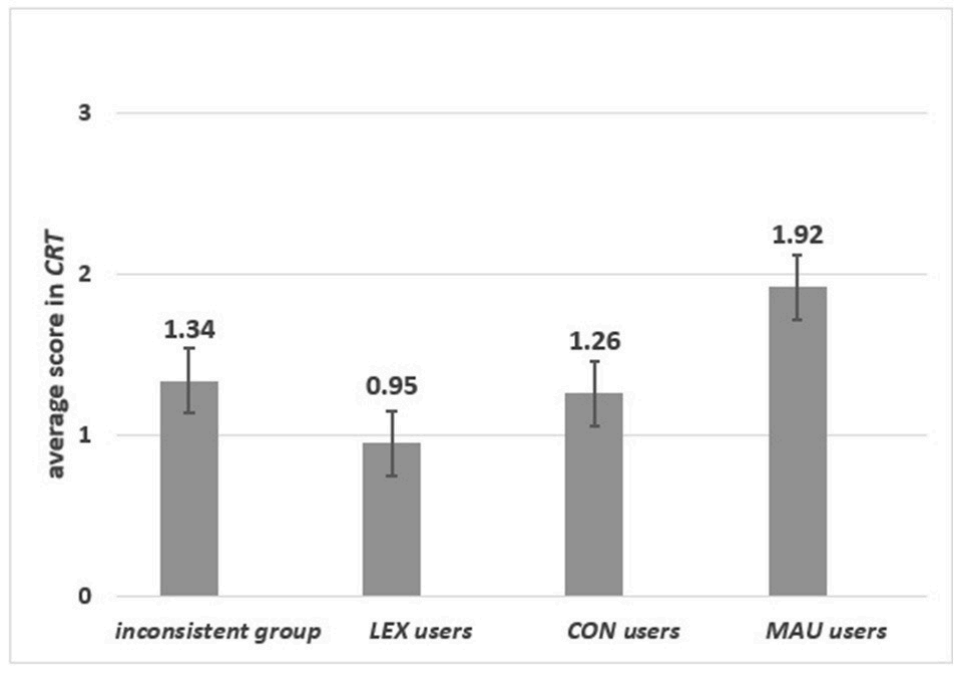
The average scores in *CRT* for respondents who applied various strategies.

There were significant differences among groups of specific strategy users in the *CRT* score [*F*_(3, 193)_ = 5.22 *p* = 0.002, η_p_^2^ = 0.075]. As can be seen from Figure 4, *MAU*-users scored significantly higher than the inconsistent group (*p* = 0.004), *LEX*-users (*p* = 0.001) and *CON*-users (*p* = 0.006).

**Figure 5:**
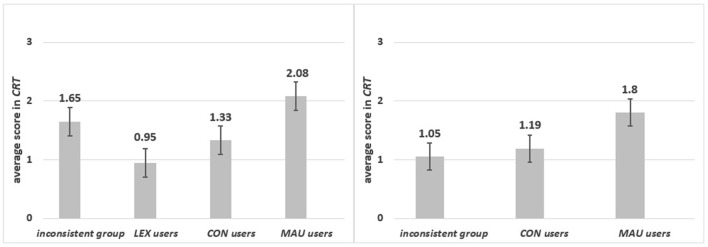
The average scores in *CRT* for respondents who applied different strategies in specific **(left panel)** and in abstract **(right panel)** content.

*Group 1: Specific content*. As can be seen from Figure 5 left panel, *MAU, CON* and *LEX* users and the inconsistent group differed in the *CRT* score [*F*_(3, 97)_ = 3.965, *p* = 0.01, η_p_^2^ = 0.109]. The lowest mean of correct responses was observed for *LEX*-users, who significantly differed from *MAU*-users (*p* = 0.001). *MAU*-users scored higher than *CON*-users (*p* = 0.04) but not then the inconsistent group (*p* = 0.15) (see Figure 5).

*Group 2: Abstract content*. Significant differences were observed in the *CRT* score [*F*_(2, 93)_ = 4.32 *p* = 0.016, η_p_^2^ = 0.085] for *MAU*-users*, CON*-users and the inconsistent group. *MAU*-users scored highest in the *CRT* (*p* = 0.007). No significant differences between *CON*-users and the inconsistent group in the *CRT* were observed (*p* = 0.648) (see Figure 5, right panel). In contrast to the result obtained for the *REI-R* in the abstract task, where users of both strategies based on global evaluations scored higher than others, only *MAU*-users scored highly in the *CRT*. This might indicate that the *CRT* also measures numeracy skills (see Discussion), not only the dominant style of information processing. Different relations were observed in the specific task where, surprisingly, the inconsistent group received high results on the *REI-R* and in the *CRT*.

### Strategy and transitivity

The score in transitivity was compared for users of *MAU, CON* and *LEX* strategies. As expected, there were significant differences in the score in transitivity among users of specific strategies [*F*_(3, 184)_ = 12.71, *p* < 0.001, η_p_^2^ = 0.172]. Neither the interaction nor the main effect of content was found in ANOVA analysis with two between-subject factors (Strategy 4 levels: *MAU, CON, LEX*, inconsistent, and Content 2 levels: abstract and specific). Therefore, the distribution of the transitivity index was compared for users of different strategies for all respondents (see Table 4). As can be seen from Table 3, the majority of those who used *MAU* (65.6%) or *CON* (76.9%) was always transitive, whereas only 40% of *LEX*-users and 29.6% of inconsistent respondents had such preferences. These differences were statistically significant (χ2 = 35.9, *df* = 6, *p* < 0.001).

**Table 4:** The distribution of the transitivity index for users of different strategies.

**Index of transitivity**	**Strategies**			
	**Inconsistent (%)**	***LEX* (%)**	***CON* (%)**	***MAU* (%)**
5	29.6	15	5.1	4.9
6	40.8	45	17.9	29.5
7	29.6	40	76.9	65.6

The above results may account for the insignificant correlations between *DPT* measures and the index of transitivity in specific content, as reported in the previous section. 19.8% of participants used *LEX* in this content. Note that using the *LEX* doesn't exclude transitivity when only one attribute is used. Indeed, 40% of *LEX*-users always had transitive preferences. Even though this percentage is lower than for users of *MAU* and *CON*, it might obscure the relation between transitivity and *DPT* measurements with specific content.

### Indexes of rationality and applied decision strategies

From the results on the three indexes of rationality and applied strategies, it appears that both *DPT* measurements and transitivity are related to applied strategies. Those who scored highly on the *REI-R* used all relevant information, making specific and abstract choices based on global evaluations in both studies. This, in turn, led to transitive preferences. Furthermore, those who scored highly in the *CRT* used global evaluation based on additive integration of information. Even though *CON*-users scored lower in the *CRT*, they had transitive preferences. This might indicate that their the *CRT* score resulted from low numeracy skills rather than from information processing.

As mentioned earlier, exceptions were participants who switched among strategies in the specific content. They scored highly on the *REI-R* and in the *CRT*. This might indicate that when people face undesirable trade-offs, they try to avoid conflict. To achieve this, they are more prone to use non-compensatory rules, either *CON* or *LEX*, or switching between them. In the case of *LEX*, they also may use more than one attribute. The poor transitivity of preferences within this group lends support to this interpretation.

## Discussion

One aim of these studies was to check the relation between *DPT* measurements and the transitivity of preferences. It was found that people who obeyed transitivity scored highly on the *REI-R* and also in the *CRT*. This is an important and new result, which implies that both measures are good predictors of respect for transitivity. Even though both *DPT* measurements were very frequently used in previous studies (e.g., Ayal and Hochman, 2009; Cokely and Kelley, 2009; Pachur and Galesic, 2013; Graffeo et al., 2015), to our knowledge such a direct test has not been reported. One example of an indirect test is the research by Ayal et al. (2015). In Experiment 1, they found that the number of biases made could be predicted from the score on the *REI* and from the induced thinking mode. In Experiment 2, they found that: (1) an induced analytical thinking mode improved transitivity in analytical tasks, and (2) an induced intuitive thinking mode improved transitivity in intuitive tasks. However, in Experiment 2 the *REI* wasn't used. Therefore, in neither of these studies was the direct relation between the *REI* and transitivity checked. In case of the *CRT*, Primi et al. (2016) checked the relation between scores in the *CRT* and the transitivity of inferences, i.e., the validity of conclusions in light of given premises. They found not significant correlation with the *CRT* (*r* = 0.24, *p* = 0.67, *N* = 59). However, the transitivity of inferences should not be confused with the transitivity of *preferences*. For example, one prominent difference is that the transitivity of preferences depends on sensitivity to differences in scores on attributes (Tversky, 1969).

It should be underlined that the positive relation between scores on the *REI-R* and the *CRT* and the transitivity of preferences was observed for abstract choices only. The key advantage of abstract frames is that they reduce conflict of values. Therefore, people perceive choices as logical tasks: experience, knowledge about the world, beliefs and other factors do not disturb decision processes. So, System 1 is not activated. For example, in an abstract frame one has to accept the importance of the attributes given in the instruction since one has neither experience nor knowledge about the situation or its attributes. So a frame of this kind should be the best experimental condition to examine individual differences in the dominant style of information processing, what should result in the transitivity of preferences These results are in line with those reported by Toplak et al. (2011).

Respondents who had transitive preferences in abstract choices, also scored highly on the *REI-R* and in the *CRT*. Lack of such relations in specific choices was most likely caused by the small fraction of *LEX*-users, who had transitive preferences even though they scored poorly in both *DPT* measurements. So for people with dominant System 2, who also meet normative criteria of rationality, content doesn't affect their rational way of decision-making. Witteman et al. (2009) obtained similar results, i.e., they found correlation between the performance in thematic and abstract Wason tasks and the score on the *REI-R*.

Another interesting result is the confirmation that the three selected strategies—i.e., *LEX, CON*, and *MAU*—are good representations of three kinds of information processing: simple heuristics, bounded rationality and normative models. As might be expected, salient differences were observed between *MAU* and *CON*-users and the others, in both the index of transitivity and the score on the *REI-R. MAU*-users, in contrast to *CON*-users, also scored highly in the *CRT*. This may reflect the difference in numeracy skills between these two groups rather than a difference in rationality measured as consistency with basic logical principles. The higher transitivity and the higher *DPT* scores of respondents who used strategies based on global evaluation is additional support for the claim that the *REI-R* and *CRT* are good measurements of individual differences in information processing in terms of rational preferences.

In light of the above conclusion, the high score on the *REI-R* and in the *CRT* found for respondents who didn't consistently use the same strategy in tasks with specific content, requires additional explanation. In these tasks, respondents had to face undesirable trade-offs that might cause problems with the weighting of attributes and therefore with the avoiding of conflict. This favors use of lexicographic rules (Beattie and Barlas, 2001) that lead to intransitivity if *JND* varies across attributes and more than one attribute is used (Tversky, 1969). They also might switch between *CON* and *LEX* strategies. Indeed, these respondents scored the lowest in the transitivity index. However, the intransitivity of specific choices might also result from sensitivity to configurations of values on attributes which are in disagreement with the independence axiom. These problems do not follow from processing information with the aid of System 1 and so are not measured by the *REI-R* or the *CRT*.

Even though most authors consider simple heuristics as belonging to the class of Bounded Rationality models, they do not test a conjunctive rule that fully reflects Herbert Simon's Principle of Satisficing. Note that a majority of research is focused on comparison of linear models with simple heuristics, whereas *CON* rule is only rarely investigated. In our research, an interesting finding was that the most differentiated results were obtained for *CON*-users. They scored as high on the *REI-R* as *MAU*-users, but in the *CRT* they scored as poorly as *LEX*-users. This might point to the willingness of *CON*-users to be rational, which is reflected in self-declarative measurements. However, this willingness is not the same as numeracy skills. On the other hand, *CON*-users had an equally high index of transitivity of preferences as *MAU*-users. This supports the thesis that in every-day decisions, making satisficing choices is as rational as optimization. In contrast, selective and partial information processing does not guarantee transitivity. Only 40% of *LEX*-users respected the transitivity axiom. They also had low scores on the *REI-R* and in the *CRT*.

As mentioned in the Introduction, the fundamental assumption in decision-making is that rational choice requires a global evaluation of each option and the additive integration of information. The alternative perspective suggested here might be that choices based on global evaluation and the Satisficing Principle, instead of averaging, are also rational. Linear models lead to the optimization for the prize of trade-offs, whereas a conjunctive rule that doesn't require trade-offs leads only to satisficing decisions. So both have advantages and disadvantages in terms of accuracy. Thus, there are no reasons to treat linear models as the single criterion of rational choice.

Our results support this view. Respondents who had transitive preferences used either averaging (31%) or the *CON* rule (19.8%), so they met the normative criterion of rationality. Moreover, they also scored highly on the *REI-R*, i.e., declared a willingness to solve problems in an analytical way. Both findings suggest that users of the *CON* rule met both objective and subjective criteria of rationality.

However, the higher score in the *CRT* found for *MAU*-users compared to *CON*-users is not in agreement with this conclusion. This may have two explanations. First, the *CRT* measures not only the dominant style of information processing but also numeracy skills-*CON*-users had lower such skills than *MAU*-users. Second, the *CRT* may be a stronger test of analytical thinking than a self-declarative measurement such as *REI-R*, as suggested by Toplak et al. (2011). Therefore, the findings that users of *CON* had transitive preferences and scored highly on the *REI-R* but not in the *CRT* might be interpreted as the difference between the concepts of rationality and Bounded Rationality. To check this hypothesis, one should investigate which cognitive abilities are related to the use of averaging vs. the Satisficing Principle as the criterion for choice. In particular, previous research was limited to a comparison between simple heuristics and linear models, forgetting Bounded Rationality idea to which they refer often.

Finally, since there is a relation between transitivity and System 2 it would be interesting to investigate whether people consider either averaging or satisficing as an effective tool for reaching accurate decisions and if so, what situational factors reinforce the tendency to use either strategy.

## Ethics statement

This study was carried out in accordance with the recommendations of the Ethics Committee of University of Social Sciences and Humanities with informed consent from all subjects. The study was conducted online and all subjects gave informed consent in accordance with the Declaration of Helsinki. The protocol was approved by the Ethics Committee of University of Social Sciences and Humanities.

## Author contributions

PS and JS developed the experimental design, collected, analyzed and interpreted the data. PS and JS wrote the first draft of the manuscript. Both authors participated in drafting the article and made critical revisions. Both authors approved the final version of the paper.

### Conflict of interest statement

The authors declare that the research was conducted in the absence of any commercial or financial relationships that could be construed as a potential conflict of interest.

## Footnotes

^1^Recently, Primi et al. (2016), investigated the relation between the *CRT* and transitivity of inferences, i.e., validity of conclusions in light of premises (see section Discussion).

^2^No hypotheses were formulated for Experiential subscale of the *REI* because there are many findings indicating that the *REI-R* is a better predictor of actual rational behavior.

^3^In this heuristic, the cue validity, i.e., its predictive accuracy, determines the attributes' order.

^4^there are several versions of the *rei* (i.e., the *rei-40*, the *rei-24* by pacini and epstein, 1999; 10-item version by epstein et al., 1996). the *rei-24* was used in two studies presented here, because of its high internal consistency (ayal et al., 2012).

^5^In Group 2, where attributes had no modalities, the choice of an option with a score close to the maximum for either attribute was considered to be a *proxy* for *LEX*.

^6^The number of intuitive answers in the *CRT* was negatively correlated with the transitivity of preferences for abstract choices (−0.157, *SE* = 0.080, *p* = 0.05, the lower −0.314 and upper −0.0002 bounds) but not for specific ones (−0.025, *SE* = 0.075, *p* = 0.74, the lower −0.173 and upper 0.124 bounds).

^7^Eight participants who used *LEX-like* strategy were omitted.
