# Supplementary material for: Measurements of Rationality: Individual Differences in Information Processing, the Transitivity of Preferences and Decision Strategies
Source: Front Psychol. 2017 Oct 18;8:1844. doi: 10.3389/fpsyg.2017.01844 (PMC5651814; doi:10.3389/fpsyg.2017.01844)
Supplement: Supplementary file 1 [file DataSheet1.pdf]

Appendix 1.

The 10 choices out of 12 were accepted on the basis of comparison of the probability of such sequence when a given strategy is applied with the probability of the random sequence. For example, if there are 12 choices that favor the *CON* strategy, a perfect decision maker who applies *CON*, should make 12 choices in line with this strategy. However, an imperfect one makes mistakes and would make less such choices, e.g. 10 or 9. Now the question is: if a person makes *M* choices consistent with strategy *X*, what is the probability, $P_{(X)}$, that s/he was applying strategy *X*? The answer follows from binomial distribution[1] and depends on the assumed probability of error. In Figure 1 (black line), such probabilities are given for all possible number of choices consistent with *CON* (from 0 to 12), allowing the probability of error 0.2.

As can be seen from Figure 1, the most probable number of choices consistent with the *CON* strategy when the probability of error is 0.2 is 10. As the number of choices in line with *CON* decreases, the probability also decreases. This is in agreement with intuition.

Contrary to intuition, the probability of choosing 12 or 11 times in line with *CON* is also lower than choosing 10 times because a possibility of error was allowed. However, this does not imply that making all 12 choices according to *CON* makes this strategy less probable than its alternatives. To assign a strategy to a participant, this probability has to be compared with the probability that choices were made at random. Random choice should be considered a universal, reference point that always needs to be convincingly rejected. In the example considered here, when two options are selected at random, both have the same probability to be chosen, i.e. the

---

[1] See e.g. https://www.easycalculation.com/statistics/binomial-distribution.php for an interactive binomial calculator. In the notation adopted here, the number of events is N, the number of success is M and the probability of success is (1 - probability of error).

probability of error and the probability of success are both 0.5. The grey line in Figure 1 represents the probability that choices are random. The shape of the probability curve for random choice agrees with our intuition. The most probable outcome is the one in the middle - 6 out of 12. As *M* increases or decreases, departing from these numbers, the probability symmetrically decreases.

Finally, the probabilities of choosing according to the strategy of interest and at random need to be compared. This was done by taking their ratio. This ratio tells us what the odds were that a respondent was using strategy *X* rather than choosing at random. The probability of 10 *CON* choices out of set of 12 (0.8 assumed success) to the probability of 10 random *CON* choices out of 12 (0.5 assumed probability of success) is: 0.283/ 0.016= 17.5929. Thus, this event is significantly different from a random event. Note, that this changes dramatically, when we talk about series of 8 choices of 12: 0.133/0.121=1.0995. On the basis of this reasoning the criterion of 10 out of 12 choices was accepted for assigning a strategy.

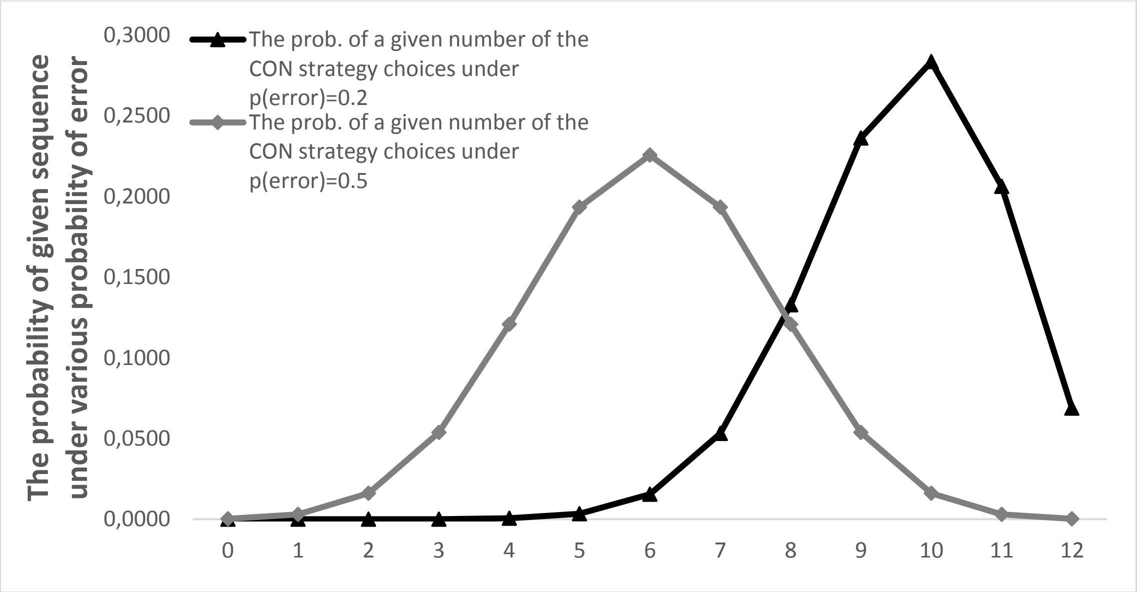

Figure 1. The probability of a given number of *CON* choices under various accepted probabilities of error.
